# Early diagnosis of occult pulmonary embolism secondary to lower limb fractures: summary of 18 cases

**DOI:** 10.3389/fmed.2024.1355030

**Published:** 2024-05-30

**Authors:** Luqin Di, Zheng Chen, Xiaoyan Wang, Jiao Zhang, Jing Zhang, Junqin Ding

**Affiliations:** Department of Orthopaedic Surgery, Third Hospital of Hebei Medical University, Shijiazhuang, Hebei, China

**Keywords:** lower limb fractures, deep vein thrombosis, diagnose, occult pulmonary embolism, orthopaedic

## Abstract

**Objectives:**

Pulmonary thromboembolism is a severe but probably underdiagnosed disorder. Patients with lower limb fractures are at high risk for pulmonary thromboembolism. This study aimed to demonstrate the early identification strategies for occult pulmonary thromboembolism.

**Methods:**

From January to December 2022, 18 patients diagnosed with pulmonary thromboembolism were reviewed for this study. Data on patients’ demographics, laboratory test results, and radiographic findings were collected. Finally, the data was analyzed.

**Results:**

Eighteen patients with lower limb fractures were included in this study. All of them present different symptoms, including 12 cases (12/18, 66%) of unexplained decrease in oxygen saturation; 16 patients had deep vein thrombosis in the lower limbs, with nine cases involving proximal veins and seven involving distal veins. One patient had an antithrombin III level of 35%. Thirteen cases were diagnosed with pulmonary embolism using CT pulmonary angiography. Four patients had pulmonary embolisms incidentally detected during coronary CT angiography, and one patient during aortic CT angiography.

**Conclusion:**

Patients with lower limb fractures showing chest tightness and unexplained decrease in finger pulse oxygen levels should be assessed for pulmonary thromboembolism. Simultaneously, selecting appropriate diagnostic tools is essential to guaranteeing quick and accurate diagnosis.

## 1 Introduction

Pulmonary thromboembolism (PTE) is a common yet severe complication in orthopedic trauma patients, typically caused by the dislodgment of deep vein thrombosis (DVT) in the lower limbs. Patients with traumatic orthopedic injuries have a higher risk of developing DVT because of reduced blood flow during immobility and heightened blood coagulation post-injury, which also increases the likelihood of pulmonary PTE. The yearly incidence rate of PTE in China is 0.1% (1), and approximately 300,000 instances of PTE occur annually in six European Union nations (2), with a mortality rate of over 20% (3). PTE has emerged as a significant global burden.

Pulmonary thromboembolism often manifests as dyspnea, chest pain, hemoptysis, syncope, and other related symptoms in clinical settings. However, in some instances, patients may exhibit no manifestations or experience mild and transient symptoms, which can easily lead to missed diagnosis and misdiagnosis. We call it occult pulmonary embolism (4). Undiagnosed and untreated occult pulmonary embolism can lead to pulmonary artery thrombosis organization and pulmonary vascular remodeling, leading to vascular stenosis or occlusion, which can cause pulmonary arterial hypertension (5). Additionally, occult pulmonary embolism can precede more severe embolisms, increasing the risk of recurrence and the potential for fatal embolism (6, 7).

Currently, the prompt identification of PTE encounters obstacles. PTE can be easily neglected or misdiagnosed due to its nonspecific symptoms and variable levels of severity. Relying solely on clinical symptoms for diagnosis is unreliable and still requires imaging equipment. Asymptomatic individuals may have overlooked the existence of PTE due to not undergoing further examinations. Researchers have found that 72% of asymptomatic DVT patients develop PTE after undergoing computed CT pulmonary angiography (CTPA) testing (8). CTPA is an effective diagnostic tool for detecting PTE. However, its application is restricted by its high cost, risk of radiation exposure, and probable adverse reactions to contrast agents, limiting its widespread clinical utility. On the other hand, patients with mild symptoms may be misdiagnosed as other illnesses and may not receive an early diagnosis. A study found a 33.5% delayed diagnostic rate for PTE (9). Therefore, promptly identifying patients with PTE through precise clinical evaluation is essential. An analysis was conducted on the case data of 18 patients with occult pulmonary embolism found in lower limb fracture patients treated at our institution between January and December 2022 to offer guidance for clinical care.

## 2 Materials and methods

### 2.1 General information

This retrospective study was carried out in a tertiary university hospital. From January to December 2022, the hospital admitted 66,595 patients, of which 180 received a diagnosis of PTE, and 18 were included in this study. The approval for this study was obtained from the hospital’s ethics committee. The inclusion criteria were as follows: ① The patients were diagnosed with lower limb fracture through X-ray or CT scans; ② PTE was diagnosed through CT angiography during hospitalization. The positive criterion was a filling defect in the pulmonary artery. Occult pulmonary embolism refers to cases where symptoms such as difficulty breathing, chest pain, hemoptysis, and hypotension are absent or mild and persistent symptoms are present. DVT was diagnosed by color doppler ultrasound. The exclusion criteria were as follows: ① people with a history of venous thromboembolism; ② concurrent malignant tumors; ③ severe liver and kidney failure; ④ incomplete case data.

### 2.2 Data collection

The intrinsic characteristics of the patient, encompassing their gender, age, stature, mass, mechanism of injury, and comorbidities, were fastidiously documented. The laboratory parameters, including blood routine examination, biochemical composition, and blood coagulation, were collected. Moreover, the clinical manifestations exhibited by the patients, alongside the results derived from various imaging examinations, were also documented.

## 3 Results

### 3.1 General information

Among the group of 18 patients diagnosed with occult pulmonary embolism secondary to lower limb fractures, there were eight male and ten female individuals. The age ranged from 42 to 81 years, with an average age of (65.11 ± 11.31) years. Examining their body mass index (BMI), the values varied between 17.58 to 35.15 kg/m^2^, with an average BMI of (26.58 ± 3.98) kg/m^2^. Notably, six patients had a BMI exceeding 28 kg/m^2^.

The causes of these fractures varied: 10 cases resulted from accidental falls, 5 cases arose from traffic accidents, 1 case was attributed to a high-altitude fall, 1 case stemmed from heavy object impact, and 1 case was a consequence of machine strangulation. Regarding the types of fractures sustained, there were 5 open fractures and 13 closed fractures. As for the specific fracture sites, the following distribution was discovered: 7 patients suffered hip fractures, five individuals experienced femur fractures, and another five sustained tibiofibular fractures. Additionally, one patient endured multiple fractures encompassing the left tibiofibular, left patella, inner ankle, and right pubic branch. In terms of laterality, 9 patients encountered fractures on their right lower limbs, 6 patients were affected on their left lower limbs, and 3 patients underwent fractures on both of their lower limbs simultaneously (Table 1).

**TABLE 1:** Clinical data of case series.

Case no.	Sex	Age	BMI	Fracture sites	Diagnosis of PTE	Time of PTE diagnosis	Clinical manifestations	Site of DVT
1	F	53	25.39	Tibiofibular fracture	CTPA	9 days after surgery	Decrease in oxygen saturation, fever	Popliteal
2	M	81	26.89	Hip fracture	CTPA	5 days after admission	decrease in oxygen saturation	femoral
3	F	69	22.46	Tibiofibular fracture	CTPA	4 days after surgery	Decrease in oxygen saturation	Tibial
4	M	53	27.68	Tibiofibular fracture	CCTA	7 days after surgery	Chest distress, cough	Popliteal
5	M	42	29.41	Tibiofibular fracture	CTPA	4 days after admission	Chest distress, cough, decrease in oxygen saturation, fever	/
6	F	54	27.55	Multiple fracture	CTPA	1 day after surgery	Decrease in oxygen saturation	Tibial
7	F	73	23.88	Femur fracture	CCTA	8 days after surgery	Chest distress	Intermuscular
8	F	70	23.44	Femur fracture	CTPA	10 days after surgery	Decrease in oxygen saturation	femoral
9	F	69	31.44	Hip fracture	CTPA	9 days after admission	Chest distress	Femoral
10	F	72	25.71	Hip fracture	CCTA	9 days after admission	Decrease in oxygen saturation	Popliteal
11	M	74	30.42	Hip fracture	CTPA	12 days after admission	Decrease in oxygen saturation	Popliteal
12	M	68	24.49	Hip fracture	aortic CT angiography	3 days after surgery	Decrease in oxygen saturation, chest pain	/
13	M	45	24.22	Femur fracture	CTPA	2 days after surgery	Decrease in oxygen saturation	Intermuscular
14	F	72	29.29	Hip fracture	CTPA	5 days after admission	Chest distress, decrease in oxygen saturation	Intermuscular
15	F	73	17.58	Hip fracture	CTPA	1 day after surgery	Decrease in oxygen saturation	Intermuscular
16	M	59	29.22	Tibiofibular fracture	CTPA	2 days after surgery	Hemoptysis	Popliteal
17	M	67	24.22	Femur fracture	CCTA	7 days after admission	Chest distress	Intermuscular
18	F	78	35.15	Femur fracture	CTPA	3 days after admission	Decrease in oxygen saturation	Femoral

F, female; M, male; CTPA, computed tomography pulmonary angiography; CCTA, coronary CT angiography; PTE, pulmonary thromboembolism; DVT, deep venous thrombosis; BMI, body mass index.

### 3.2 Laboratory parameters

The plasma concentrations of D-dimer in all 18 patients exhibited an initial surge following admission, with values ranging from 1.32 to 67.97 mg/L and a median measurement of 11.68 mg/L. Subsequently, during the occurrence of PTE, these levels ranged from 0.61 to 37.47 mg/L, with a median of 2.23 mg/L, all yielding positive results following age correction. The D-dimer level surpassed the admission measurement in six patients near the onset of PTE, while it diminished in twelve patients. Another indicator, antithrombin III, was found to be 35% with a hereditary deficiency in one patient. His brother had a history of postoperative pulmonary embolism, and his children all had outpatient tests for antithrombin III of about 30%.

### 3.3 Occurrence of DVT in lower limbs

In these cases, 10 patients (56%) showcased the presence of DVT in the lower limbs via the use of ultrasonography prior to the diagnosis of PTE. Specifically, there were 4 occurrences of thrombosis in the femoral vein, 4 in the popliteal vein, 1 in the posterior tibial vein, and 1 in the intermuscular vein of the lower leg. Furthermore, 6 patients (39%) did not exhibit DVT prior to the detection of PTE; However, subsequent ultrasound examinations following the onset of PTE confirmed the existence of thrombi. These included one case of popliteal vein thrombosis, one case of posterior tibial vein thrombosis, and four cases of intermuscular vein thrombosis in the lower leg. Remarkably, two patients (11%) did not manifest lower limb DVT during both pre and post-PTE ultrasound assessments.

Except for one patient suffering from subcapsular hematoma and two individuals afflicted by craniocerebral trauma, all other patients received anticoagulant therapy utilizing low molecular weight heparin (LMWH) commencing on their first day of hospital admission. Subsequently, four patients underwent the placement of temporary inferior vena cava filters as an adjunct to pharmacological anticoagulation upon the identification of DVT.

### 3.4 Occurrence of PTE

Clinical manifestations: 18 patients had different degrees of symptoms, including 12 cases (66%) of unexplained decrease in oxygen saturation, 7 cases (38%) of chest distress, 2 cases (11%) of fever, 2 cases (11%) of cough, 1 case (5%) of chest pain, and 1 case (5%) of hemoptysis.

Time of PTE: 3 cases occurred after a change in body position (including turning over, taking a bedside X-ray, and transferring from bed to wheelchair), 1 case after forceful defecation, 1 case after underground activities, and the remaining 13 cases were not recorded.

Diagnosis of PTE: 13 cases were diagnosed with pulmonary embolism by computed tomography pulmonary angiography (CTPA); 4 patients considering coronary artery disease found pulmonary embolisms accidentally by coronary CT angiography (CCTA); 1 patient with chest pain evaluating aortic lesions was found to have a dissection of the aorta combined with pulmonary embolism on aortic CT angiography.

Time of PTE diagnosis: 8 cases occurred preoperatively, and 10 points occurred postoperatively, with a mean of (6.8 ± 3.1) days after admission and (4.7 ± 3.5) days after surgery.

### 3.5 Pulmonary embolism site

There were 8 cases of embolism affecting the right pulmonary artery and its branches, comprising 3 occurrences of partial blockage in the main pulmonary artery, 3 in the lobar pulmonary artery, and 2 in the segmental pulmonary artery. In addition, there were 4 cases of embolism observed in the left pulmonary artery and its branches. This included 1 case of incompletely obstructed main pulmonary artery, 1 case affecting the lobar pulmonary artery, and 2 cases occurring in the segmental pulmonary artery. Furthermore, there were 6 instances of embolism witnessed in both pulmonary arteries and their branches, which encompassed 2 cases of incomplete obstruction in the main pulmonary artery and 4 cases impacting the additional pulmonary arteries.

### 3.6 Typical cases

**FIGURE 1:**
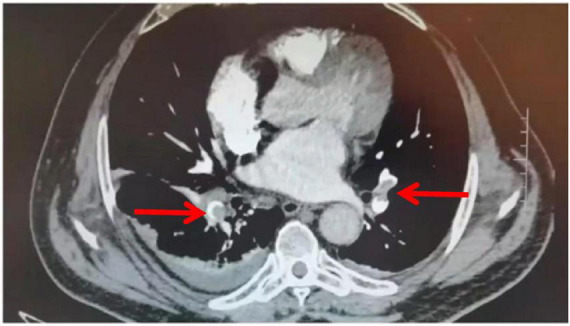
CTPA image of the patient before treatment (arrow indicates the site of embolism).

**FIGURE 2:**
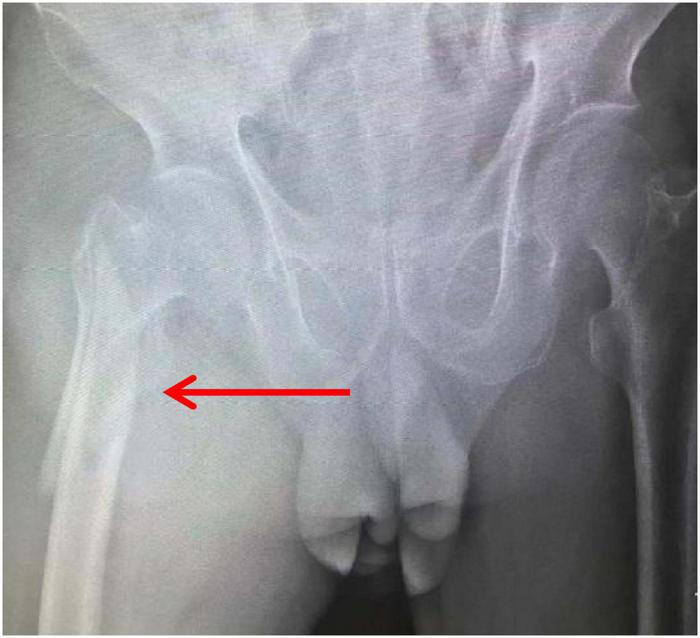
X-ray.

**FIGURE 3:**
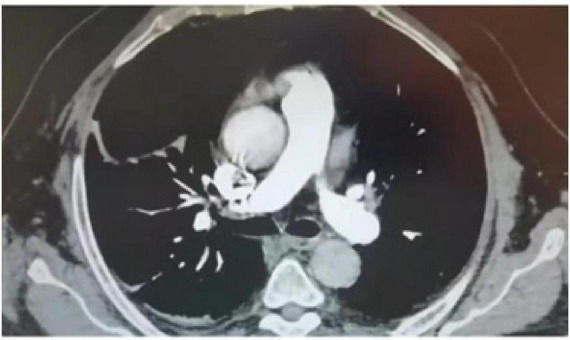
CTPA image of the patient after treatment.

An 81-year-old male patient was hospitalized for right hip pain and restricted movement resulting from a fall that occurred 8 h ago. The X-ray revealed fractures in the right femoral trochanter and proximal femur. The patient has a history of high blood pressure. He received low-molecular-weight heparin treatment while in the hospital. The D-dimer level measured 13.79 mg/L. On the second day of admission, the patient underwent a bedside X-ray and turned over, causing a drop in finger pulse oxygen levels from 98% to 94%. The heart rate was 89 bpm, and the blood pressure was 161/74 mmHg. The patient did not experience any discomfort. An immediate deep vein ultrasound examination of both lower extremities revealed thrombosis in the right superficial femoral vein, popliteal vein, posterior tibial vein, and intermuscular vein of both calves. CTPA revealed the presence of blood clots in both lungs. After being moved to the pulmonary department for standardized anticoagulant treatment, the patient experienced notable improvement. Figures 1–3 display the imaging data.

## 4 Discussion

### 4.1 Enhance the awareness of PTE

PTE is characterized by the classic symptoms of shortness of breath, chest pain, and coughing up blood. The lung receives oxygen from the pulmonary artery, bronchial artery, and intra-alveolar gas diffusion. Less than 15% of patients experience all three oxygen supplies simultaneously (10). Patients commonly report chest distress, which can be mistaken for heart illness. PTE is characterized by its abrupt onset and is frequently linked to DVT or similar risk factors, in contrast to heart disorders. Seven patients in our study experienced chest discomfort and had various risk factors, including deep vein thrombosis, trauma, surgery, and prolonged bed rest. Based on the Wells Simplified score, all seven patients were deemed probable for pulmonary thromboembolism, but only four were confirmed with computed tomography pulmonary angiography. The remaining cases were incidentally discovered using PTE during the coronary heart disease screening, suggesting a potential lack of knowledge on PTE. Thus, when patients exhibit the symptoms above, it is crucial to investigate their medical history thoroughly, assess the presence of deep vein thrombosis or associated risk factors, and be vigilant for the potential occurrence of pulmonary embolism instead of solely focusing on cardiac illness.

In these cases, the primary indication of PTE was a significant decrease in pulse oxygen saturation (about 66%), with blood gas measurement showing hypoxemia. Research has shown that 14.3% of individuals with hypoxemia due to COVID-19 experienced PTE (11). This highlights the need to enhance PTE awareness, consider the predictive significance of hypoxemia, and routinely screen hypoxemic patients for PTE.

### 4.2 Pay attention to high-risk populations

PTE is mainly caused by emboli originating from DVT in the lower limbs, and individuals with DVT are at a high risk of developing occult pulmonary embolism. Research indicates that 32–72% of patients with DVT experience covert pulmonary embolism (6, 8). In our study, DVT was found in 16 cases, accounting for around 89% of the cases. Therefore, screening patients with DVT for risk is crucial to detect occult pulmonary embolism early.

Research indicates that the occurrence of occult pulmonary embolism is more common in patients with proximal deep vein thrombosis (including popliteal vein thrombosis and proximal vein thrombosis) compared to those with distal deep vein thrombosis (calf muscle venous plexus thrombosis) (6, 12). In our cases, the occurrence of proximal DVT in patients with PTE is 50%, which is greater than the incidence of distal DVT (39%), in line with prior research. This could be due to the proximal vein’s increased thickness, leading to larger blood clots and a greater likelihood of a dislodged embolus blocking the pulmonary artery. Distal deep vein thrombosis is generally believed to have a lower likelihood of causing pulmonary thromboembolism, and there is no agreement on how it should be treated. The ACCP guidelines recommend continuous imaging examination rather than anticoagulant therapy in patients without severe symptoms or thrombotic extension risk factor (13). Seven patients in our group with distal DVT experienced PTE, suggesting that current treatment for distal DVT may be inadequate. There is a 9% possibility that distal DVT may extend to the proximal end despite anticoagulant treatment (14). The identified thrombus could be a residual part that has moved to the lung, putting distal DVT at risk for PTE, which is a significant issue.

Drug prophylaxis is the most crucial, practical, measurable, and widely recommended preventive intervention in guidelines. LMWH is frequently utilized in clinical settings. However, certain aspects require attention. ① The dosage of the anticoagulant may be inadequate. The guidelines advocate adjusting the dosage of LMWH based on body weight. LMWH is available in several dose forms, primarily pre-filled containers that are difficult to divide based on body weight. The patients in our situation weigh between 45 and 100 kilograms. The patients received Low Molecular Weight Heparin Sodium Injection (0.4 ml: 4250 IUaXa), Nardrheparin Calcium Injection (0.4 ml: 4100AXaIU), and Enoxapinana Sodium Injection (0.6 ml; 6000AxaIU), potentially resulting in inadequate dosage for individuals with higher body weight; ② “Heparin resistance” issue (15). The anticoagulant properties of LMWH are reliant on antithrombin III levels. Low levels of antithrombin III can decrease individuals’ responsiveness to LMWH. Antithrombin III levels were at 35% in one patient, and the use of LMWH did not achieve adequate anticoagulation. Therefore, it is advisable to choose suitable anticoagulants based on individual variations in clinical settings.

D-dimer is a highly sensitive marker for PTE. Research shows that elevated d-dimer levels within 24 h of admission are an independent risk factor for occult pulmonary embolism (16). Therefore, D-dimer can act as a vital indicator for pulmonary embolism. However, in this study, all patients tested positive for d-dimers. Both elderly patients and trauma might lead to increased levels of d-dimer, which reduces its diagnostic effectiveness. Therefore, it is crucial to incorporate extra inspections and consistently monitor d-dimer.

### 4.3 Select appropriate diagnostic tools

PTE can be classified into three categories based on the degree of danger (17): high-risk, intermediate-risk, and low-risk. High-risk individuals have unstable hemodynamics, mainly manifested by hypotension and shock, and are prone to sudden death. Moderate-risk individuals display stable hemodynamics yet present with right heart dysfunction or myocardial cell impairment. In the acute phase, it is estimated that 3–15% of patients may experience deterioration or even succumb to death. Low-risk individuals often do not have the above symptoms. CTPA is the preferred diagnostic method for PTE, but different diagnostic strategies should be adopted based on patient risk stratification. High-risk patients with PTE often do not have the conditions to undergo CTPA, and during transportation, the removal of large emboli can easily lead to sudden death. Ultrasound echocardiography can be performed beside the bed to assist in diagnosis. In the author’s hospital, a patient experienced a decrease in blood oxygen saturation from 100% to 96% under general anesthesia during surgery. Subsequently, echocardiography was administered at the patient’s bedside, disclosing the presence of an immense clot within the right atrium, incongruously drifting in tandem with each contraction of the cardiac muscle. After treatment, the patient recovered well.

All 18 cases in this group are non-high-risk patients with stable hemodynamics, and D-dimer is positive. According to the guidelines, CTPA is recommended for these patients. In this group, 13 cases were diagnosed by CTPA, and 4 cases were confirmed by CCTA. CCTA has certain limitations, however. Given that the CCTA scan occurs after the CTPA scan and has a relatively restricted scanning field, it fails to provide a precise and comprehensive evaluation of the pulmonary artery’s contrast media filling status. Consequently, post-processing reconstruction of the pulmonary artery or its branches becomes unattainable, leading to potential missed diagnoses of PTE. Notably, one patient exhibited no abnormalities in their CCTA. However, a local filling defect was discovered in the right middle lobe pulmonary artery during the subsequent CTPA on the second day. This necessitated two separate arteriograms, causing significant inconvenience for the patient. Thus, conducting a multi-site CTA examination with a single contrast injection is imperative. However, achieving this with traditional CT imaging proves challenging due to scanning time limitations and the contrast agent’s metabolism. Advancements in CT hardware equip some cutting-edge machines with the capability to perform a comprehensive cardiovascular scan in one go. It enables simultaneous acquisition of CTA imaging data from various regions, including the aorta, coronary artery, and pulmonary artery, thereby facilitating swift and accurate differential diagnoses.

## 5 Limitations

This study has certain limitations. Firstly, this is a retrospective, non-comparative study that lacks a control group, making it difficult to evaluate the role of confounding factors. However, the viewpoints we propose can provide a reference for clinical practice. Additionally, the sample size is also small, and the research findings are confined to the hospital period without long-term follow-up.

## 6 Conclusion

PTE may occur in both proximal venous thrombosis and distal venous thrombosis. When a patient with lower limb fracture presents with chest tightness and decreased oxygen saturation, the possibility of PTE should be considered.

## Data availability statement

The original contributions presented in this study are included in the article/supplementary material, further inquiries can be directed to the corresponding author.

## Ethics statement

The studies involving humans were approved by the Third Hospital of Hebei Medical University. The studies were conducted in accordance with the local legislation and institutional requirements. The participants provided their written informed consent to participate in this study.

## Author contributions

LD: Writing – original draft, Project administration. ZC: Writing – original draft, Writing – review and editing. XW: Data curation, Investigation, Writing – original draft. JiaZ: Data curation, Investigation, Writing – original draft. JinZ: Formal analysis, Data curation, Writing – original draft. JD: Writing – original draft, Writing – review and editing, Supervision, Validation.
